# Selected indigenous drought tolerant rhizobium strains as promising biostimulants for common bean in Northern Spain

**DOI:** 10.3389/fpls.2023.1046397

**Published:** 2023-03-29

**Authors:** Arantza del-Canto, Álvaro Sanz-Saez, Anna Sillero-Martínez, Eider Mintegi, Maite Lacuesta

**Affiliations:** ^1^ Department of Plant Biology and Ecology, Pharmacy Faculty, University of the Basque Country, Paseo de la Universidad n° 7, Vitoria-Gasteiz, Spain; ^2^ Department of Crop, Soil, and Environmental Sciences, Auburn University, Auburn, AL, United States

**Keywords:** growth, inoculation, native strains, nodulation, *Phaseolus vulgaris*, phenology alteration, symbiosis, water relations

## Abstract

Drought is the most detrimental abiotic stress in agriculture, limiting crop growth and yield and, currently, its risk is increasing due to climate change. Thereby, ensuring food security will be one of the greatest challenges of the agriculture in the nearest future, accordingly it is essential to look for sustainable strategies to cope the negative impact of drought on crops. Inoculation of pulses with biostimulants such as rhizobium strains with high nitrogen fixation efficiency and drought-tolerance, has emerged as a promising and sustainable production strategy. However, some commercial inoculums are not effective under field conditions due to its lower effectiveness against indigenous rhizobium strains in the establishment of the symbiosis. Thus, in the present study, we evaluated the ability to improve drought tolerance in common bean plants of different indigenous rhizobia strains isolated from nearby crop fields in the Basque Country either affected by drought or salinity. The plants in this trial were grown in a climatic chamber under controlled conditions and exposed to values of 30% relative soil water content at the time of harvest, which is considered a severe drought. From the nine bacteria strains evaluated, three were found to be highly efficient under drought (namely 353, A12 and A13). These strains sustained high infectiveness (nodulation capacity) and effectiveness (shoot biomass production) under drought, even surpassing the plants inoculated with the CIAT899 reference strain, as well as the chemically N-fertilized plants. The tolerance mechanisms developed by plants inoculated with 353, A12 and A13 strains were a better adjustment of the cell wall elasticity that prevents mechanical damages in the plasma membrane, a higher WUE and an avoidance of the phenological delay caused by drought, developing a greater number of flowers. These results provide the basis for the development of efficient common bean inoculants able to increase the yield of this crop under drought conditions in the Northern Spain and, thus, to be used as biostimulants. In addition, the use of these efficient nitrogen fixation bacteria strains is a sustainable alternative to chemical fertilization, reducing cost and minimizing its negative impact on environment.

## 1 Introduction

Drought is the most severe abiotic stress in agriculture, limiting crop growth and yield (Soureshjani et al., 2019) and due to climate change its duration and severity is expected to increase in upcoming years, especially in southern Europe (IPCC, 2021). Common bean (*Phaseolus vulgaris* L.) is the grain legume for direct human consumption of greatest production worldwide (FAO, 2021) and one of the crops with the highest nutritional value due to its high content of protein, fibers, vitamins, minerals and bioactive compounds (FAO, 2021). Unfortunately, drought is, among abiotic stresses, the most detrimental constraint of grain yield for common bean, affecting about 60% of its production areas (Polania et al., 2017; Soureshjani et al., 2019; Rai et al., 2020). Its yield is reduced between 10 and 90% depending on the frequency, duration and intensity of the drought (Soureshjani et al., 2019; Rai et al., 2020).Thereby, is an urgent need to increase common bean yield under drought conditions in order to fulfill food demands of the continuously growing global population (FAO, 1992). Consequently, maintaining food security will be one of the greatest challenges of the agriculture in the nearest future.

Soil and plant roots are colonized by a great variety of microorganisms many of which are beneficial to plants (Singh et al., 2017; Khan et al., 2020). Among them are the so-called plant growth-promoting rhizobacteria (PGPR) that inhabit rhizosphere and endo-rhizosphere of plants promoting plant growth through multiple mechanisms, such as nitrogen fixation (Verma et al., 2010; Goyal et al., 2021). Rhizobia, bacteria that perform symbiosis with legume plants, are included as PGPR mainly for their ability to fix atmospheric nitrogen through the biological nitrogen fixation (BNF) (Lindström & Mousavi, 2020). Thanks to the establishment of efficient symbiotic relationships, the legume crops productivity is increased, as well as the N content of soils, without synthetic N-fertilizers application (Weese et al., 2015; Abebe & Deressa, 2017; Torabian et al., 2019; Goyal et al., 2021), avoiding their economic and environmental costs, such as degradation of the quality of limited and scarce resources (water and soil) and increase in agricultural GHG emissions, among others (FAO, 1990; Szparaga et al., 2019; Hossain & Bakhsh, 2020). In fact, agricultural techniques that provide the necessary fertilizers from natural sources, as occurs with the establishment of legume efficient symbiotic relationships, are particularly relevant today, as a result of the large price increases experienced in recent years due to the energy crisis (Horoias et al., 2022; Sargentis et al., 2022; Statista, 2022). Common bean is considered a poor nitrogen fixer crop since it tends to establish inefficient symbiotic relationships with rhizobia species (Michiels et al., 1998). Therefore, finding rhizobium strains that are able to promote high nitrogen fixation should be a priority in order to increase yields without chemical fertilizers.

In legume plants, drought is also one of the main factors that affect more negatively the rhizobial-plant interactions and therefore the biological nitrogen fixation. Water stress affects adversely all the aspects of the legume-rhizobium symbiosis, being one of the mayor problems the rhizobia survival and persistence in the soils (Prudent et al., 2019; Santillana Villanueva, 2021; Sharaf et al., 2019; Sindhu et al., 2020). Drought also inhibits nodulation and nitrogen fixation). Few strains of rhizobia show high tolerance to water stress (Sharaf et al., 2019).

In order to maintain legume yield under water stress conditions, inoculation of pulses with highly efficient and drought-tolerant nitrogen fixing rhizobium strains has emerged as a promising sustainable production strategy. In this way, promising results have been obtained by inoculating drought-tolerant rhizobia in chickpea (Aulakh et al., 2020; Laranjeira et al., 2021), lentil (Sijilmassi et al., 2020), soybean (Igiehon et al., 2021; Omari et al., 2022), mungbean (Sapna & Sharma, 2020; Nawaz et al., 2021), pea (Prudent et al., 2019) and common bean (Figueiredo et al., 2008; Sijilmassi et al., 2020; Rodiño et al., 2021), leading to an improvement of legume drought tolerance and increasing yield under water stress. In addition, some osmotolerant rhizobial strains are also capable of surviving and nodulating efficiently under drought conditions in the soil (Fernandez-Aunión et al., 2010; Mhadhbi et al., 2011; Viti et al., 2021), and its inoculation increases the drought tolerance in legumes (Kibido et al., 2019). Mhadhbi et al. (2011) observed how the drought tolerance of common bean improved greatly after inoculating the plants with the osmotolerant strain *Ensifer meliloti* 4H41, isolated from common bean nodules from a Tunisian oasis (Mnasri et al., 2007).

The most widely used commercial rhizobium inoculum for the improvement of common bean production in the world is the Brazilian strain *Rhizobium tropici* CIAT 899 (Hungria et al., 2000). This strain is also able to nodulate a variety of other legumes and has been shown to have high tolerance to salt and drought stress (Mhadhbi et al., 2011; Sijilmassi et al., 2020) and to high temperatures (Hungria et al., 2000). Due to these characteristics, it has been used as a control or reference strain in countless common bean and soybean research (Mhadhbi et al., 2011; Cardoso et al., 2017).

However, commercial or reference inoculum may vary in effectiveness with soil type or legume genotypes. Firstly, rhizobia strains need to survive for long time in the soil in the absence of the legume (Sindhu & Dadarwal, 2000; Ruiz‐Díez et al., 2012) meaning that different geographical areas with different edaphoclimatic characteristics may not be favourable for the survival of the commercial inoculum or for the establishment of the symbiosis (Sindhu et al., 2020; Omari et al., 2022). Secondly some commercial inoculum lost effectiveness when translated from greenhouse to field due to the competition with indigenous rhizobia strains that are more effective in the establishment of the symbiosis (Kumar & Verna, 2018; Sindhu et al., 2020). Thus, usually, indigenous rhizobia are better adapted to local edaphoclimatic conditions and are more competitive with the microbiota of the specific area being more successful in nodulating the genotypes cultivated in that region (Koskey et al., 2017; Omari et al., 2022). For these reasons, we selected 8 rhizobium strains isolated from nodules of common bean cultivars grown under drought and saline field conditions in Northern Spain, with the objective of testing the effect of inoculation on different plant physiological variables related with drought tolerance, such as osmotic adjustment, cell wall elasticity adjustment, early flowering or increase in water use efficiency, among others.

Thus, the present work was based on the hypothesis that the inoculation of common bean plants with different indigenous rhizobia strains isolated under drought and saline conditions would also enhance common bean tolerance to water stress. With this aim, we studied the different drought tolerance mechanisms developed by plants after the inoculation with indigenous rhizobia isolated from local soils exposed to water and salinity stress conditions, and we selected the most efficient ones, expecting that in the future, they could be used as biostimulants to improve common bean yield under water stress in the Northern Spain.

## 2 Materials and methods

### 2.1 Biological material and growth conditions

The bacterial strains tested in this research belong to a collection of potentially drought-tolerant indigenous rhizobia cultures and to a collection of osmotolerant indigenous rhizobia cultures of the Department of Plant Biology and Ecology of the University of the Basque Country (UPV/EHU), preserved at -80°C in 50% glycerol TY medium (**Table 1**). Two rhizobium species, *Rhizobium giardinii* (343) and *Rhizobium gallicum* (353), were isolated from common bean plants grown under drought conditions in agricultural soils located in Álava (Basque Country, Spain), and six more species were isolated from common beans grown in saline soils located in Las Bardenas Reales (Navarra, Spain): *Rhizobium giardinii* (A5), *Rhizobium phaseoli* (A9), and five cultures identified as *Rhizobium etli* (A10, A11, A12 and A13) (del-Canto, 2022). All of them isolated from ecologically managed soils, with a high microbial diversity. In addition to these strains, the *Rhizobium tropici* CIAT899 strain was also tested as a reference strain of proven efficiency under drought conditions (Mhadhbi et al., 2011; Sijilmassi et al., 2020). This strain was provided by Dr. Juan Sanjuan from the Plant-Bacteria Interactions Laboratory of the Zaidín Experimental Station, CSIC (Granada, Spain).

**Table 1:** Bacterial strains selected for the study.

Code	Specie	Strain colection
343	*Rhizobium giardinii*	Potentially drought tolerant
353	*Rhizobium gallicum*	Potentially drought tolerant
A5	*Rhizobium giardinii*	Osmotolerant strains
A9	*Rhizobium phaseoli*	Osmotolerant strains
A10	*Rhizobium etli*	Osmotolerant strains
A11	*Rhizobium etli*	Osmotolerant strains
A12	*Rhizobium etli*	Osmotolerant strains
A13	*Rhizobium etli*	Osmotolerant strains

The common bean genotype selected for the assay was Arrocina de Álava, a local genotype of great economic interest, widely cultivated in Álava, Spain, and certified with the seal of quality from the Basque Country (Eusko Label, https://euskolabel.hazi.eus/es/producto-eusko-label/legumbres-del-pais-vasco/), but not studied to date.

### 2.2 Experimental design

The symbiotic performance and the drought tolerance conferred by their inoculation were tested in a pot experiment in a controlled environment grow chamber (ASL Ibercex S.A., Alcalá de Henares). Seeds were surface-sterilized by immersion in NaClO 1% (v/v) for 10 min and thoroughly washed with sterilized distilled water. The seeds were sown in 3L capacity pots, suitable for plant growth evaluations in rhizobia experiments for common beans (Obede da et al., 2020), filled with sterile perlite: vermiculite (2:1 v/v). To reduce the possibility of cross-contamination, the pots were placed in individual plastic plates and covered with thick plastic bags, all previously autoclaved (121°C, 30 min). Three seeds per pot were sown and once the seedlings emerged, only one was left, ensuring that all had a homogeneous size and vigor. Plants were grown under 14 h light photoperiod and 500 μmol photon·m^–2^ s^–1^ of light intensity with a day/night temperature of 23/18°C and 65/70% relative humidity, respectively.

The water and inoculation treatments were assigned following a completely randomized design with 10 inoculum treatments and 2 water availability treatments and replicated 8 times. The inoculation treatments were: non-inoculated plants and N-fertilized; inoculated with CIAT899 reference strain (*Rhizobium tropici*); strain 343 *(Rhizobium giardinii*); strain 353 (*Rhizobium gallicum*); strain A5 (*Rhizobium giardinii*); strain A9 (*Rhizobium phaseoli*); strain A10 (*Rhizobium etli*); strain A11 (*Rhizobium etli*); strain A12 (*Rhizobium etli*) and strain A13 (*Rhizobium etli*). The 160 pots were randomly distributed and rotated periodically on the growth chamber tables to avoid intra-chamber variability.

Throughout the plant development, the whole plant transpiration data, phenological stage, and the number of flower and leaves were recorded. The day before harvest, photosynthesis, fluorescence, SPAD and water potential data were taken from the central leaflet of full-expanded leaf. All plants were harvested at 40 days after emergence, after 25 days of drought treatment.

### 2.3 Inoculation and water stress treatment

Once sterilized, the seeds were inoculated before sowing with the nine selected bacteria according to a modified version from Sanz-Sáez et al. (2015). Summarizing, 20 mL of 10^9^cell·mL^-1^ TY medium of each strain was taken, centrifuged at 5,100 *g* for 10 min and re-suspended in 200 μL of sterile distilled water with 2% PVPP. These 200 μL water rhizobial cell suspension of 10^11^ cell·mL^-1^ concentration was added to another 50 mL of sterile water and pour it over the approximately 700 seeds and let for 24 hours, until the evaporation of water. Uninoculated seeds were also included in the experiment as a sterility control treatment. The seeds were treated as described previously for inoculated seeds but with TY medium without inoculum. In addition, the rhizosphere of each seedling was inoculated three times at 3, 7 and 11 days after emergence with 3 mL of a water rhizobial cell suspension of 10^8^ cell mL^-1^ per plant in the inoculated treatments, and with sterilized water in non-inoculated treatment (Sanz-Sáez et al., 2015).

The plants were watered at field capacity (100% relative soil water content) until the establishment of the drought. Once the drought treatment began (15 days after emergence), the well-watered plants were irrigated at field capacity, while the stressed plants were subjected to drought by completely withholding watering until reaching 30% relative soil water content. From this moment, only the transpired water was replaced to maintain the plants at this relative soil water content. The plants were irrigated three times a week, alternating water and nutritive solution to avoid salt accumulation. The inoculated plants were watered with Evans N free solution (Evans, 1974), while the uninoculated plants were watered with Evans’s solution with 10 mM KNO_3._ Thus, in the inoculated plants, as they were grown on an inert substrate watered with N free nutrient solution, all the nitrogen presented by plants came from the seed reservoir and from the plant’s nitrogen fixation (Díaz de Alcántara, 2010).

### 2.4 Phenological and growth parameters

The phenological stage, number of leaves and flowers of all plants were recorded three times a week, the watering days, from sowing to harvest. The phenological stage of all plants was determined based on the scale established by CIAT (Fernández et al., 1986).

At harvest time, the plants were separated into aerial and root biomass and different growth parameters analyzed. The area of the youngest full-expanded leaf (cm^2^) and the plant total leaf area (m^2^) were determined using a Licor 3100 area meter (LI-COR Biosciences). Leaves, stems and the full-expanded leaf were weighed separately to obtain their fresh weight (g). The dry weight (g) was obtained after drying in an oven at 80°C, a minimum of 48 hours. From these data leaf biomass (g), stem biomass (g) and shoot biomass (g), as the sum of leaf and stem biomass, were obtained.

The root systems were washed, and nodules were detached and weighed to determine the nodule fresh weights per plant (g). Then, the root dry weight (g) was obtained as explained before. Total biomass (g) was calculated adding the root biomass and shoot biomass.

### 2.5 Water relations

The whole plant transpiration (T, g H_2_O·plant^-1^) was calculated by gravimetric method by weighing each pot before and after watering and calculating the evapotranspirated water from the previous irrigation. Thus, adding all the obtained values per day, the accumulated evapotranspiration per plant was calculated. The whole plant water use efficiency (WUE, g DW·Kg^-1^ H_2_O) was calculated as the relation between the total dry matter produced and the total water transpired (Medrano et al., 2007).

The day before harvest, the leaf water potential at midday (Ψw, MPa) was measured on the full-expanded leaf´s terminal leaflet, using a pressure chamber (Skye model SKPM1400) according to the method described by Scholander et al. (1965) and modified by Mena-Petite et al. (2001). The relative soil water content (%) was determined by the gravimetric method described by Epron (1997) and modified by Miranda-Apodaca et al. (2015). Immediately after harvesting the plants, about 100 mL of humid substrate from central zone of the roots was weighed obtaining the fresh weight (g), dried at 80°C for a minimum of 48 hours and weighed again to obtain the dry weight (g). With these data, the relative soil water content was calculated as follows:


$$\text{RSWC }\left(\%\right)=\;\frac{\text{FW}-\text{DW}}{\left(\text{DW }\left(\text{FWi}/\text{DWi}\right)\right)-\text{DW}}*100$$

Where FWi was the fresh weight of each pot kept at field capacity and DWi was the dry weight of each pot kept at field capacity (droughted) of the well-watered plants.

The relative leaf water content was calculated using the gravimetric method according to Pérez-López et al. (2009a). After obtaining the fresh weight (FW, g) of three discs of 0,8 mm of diameter of the central portion of the right lateral leaflet of the full-expanded leaf, they were introduced into a Petri dish with distilled water and kept during 24 h at 4°C and in the dark (to avoid processes of respiration and photosynthesis, and thus changes of dry matter). After that, they were weighed to obtain the turgid weight (TW, g) and then dried in oven for a minimum of 8 h at 80°C to quantify the dry weight (DW, g). The relative leaf water content (RLWC) was then calculated using the following equation:


$$\text{RLWC }\left(\%\right)=\;\frac{\text{FW}-\text{DW}}{\text{TW}-\text{DW}}*100$$

The osmotic potential, (Ψo, MPa) and osmotic potential at maximum turgor, Ψo^100^ (MPa) were obtained from fresh and turgid plant material, respectively, of central zone of the full-expanded leaf´s central leaflet as described Pérez-López et al. (2009a). To obtain the turgid plant material, the leaflet was rehydrated in a Petri dish with distilled water for 24 h at 4°C in the dark. After that, in both cases, the plant material was cut in small pieces and introduced into a 0.5 mL Eppendorf tube perforated at the base that was introduced in another 1.5 mL Eppendorf tube and frozen in liquid nitrogen to achieve the rupture of the cell membranes. Then, the samples were stored at -80°C. After a few days, the tubes were defrosted and centrifuged at 13,200 *g* for approximately 5 minutes. This technique allows to separate cellular sap, which remains in the larger Eppendorf, from the solid fraction, which remains in the small one. 25 µl of cellular sap were taken and kept in a bath at 25°C, for 10 minutes moment at which the osmolarity (osmol·Kg^-1^) was measured by an osmometer (Osmomat 3000 basic, Gonotec, Germany).

The osmotic potential was calculated using the Varit Hoff’s Law according to Wyn jones & Gorham (1983):


Ψo(MPa)= −n∗R∗T

Where *n* is the osmolarity provided by the osmometer (osmol·Kg^-1^), *R* corresponds to the universal gas constant (0.0083 Kg·MPa·mol^-1^·K^-1^) and *T* is the absolute temperature of the sample (K°). The osmotic adjustment (MPa), was calculated as the difference between the mean value of Ψo^100^ of the control plants (WW) and the mean value of Ψo^100^ of plants subjected to water stress (D). The osmotic adjustment (OA) of the irrigated control plants is assumed to be zero.


$$\text{OA}={\text{Ψo}}^{100}\text{ww}-{\text{Ψo}}^{100}\text{D}$$

The dehydration (DH, MPa) of the leaves (full-expanded leaf) from six plant per treatment was estimated as the difference between the solute potential values of fresh material and the material at full turgor using following formula:


$$\text{DH}=\text{ Ψo}-{\text{Ψo}}^{100}$$

The turgor pressure or cell wall potential (Ψt, MPa), was calculated as the difference between the leaf water potential (Ψw) and osmotic water potential (Ψo).


Ψt (MPa)= Ψw−Ψo

The wall cell elastic modulus (ϵ, MPa), was estimated according to Rivelli et al. (2002), with the following formula:


$$\text{ϵ }\left(\text{MPa}\right)=\frac{\Delta \text{Ψt}}{\text{AV}/\text{V}}$$

where ΔΨt is the difference of turgor pressure (MPa) between full turgor conditions (Ψt^100^= Ψo^100^) and growth conditions (Ψt):


$$\Delta \text{Ψt}=({\text{Ψo}}^{100}={\text{Ψt}}^{100})-\text{Ψt}$$

and AV/V is the difference between the relative leaf water content in growth conditions (RLWC) and the the relative leaf water content of the fully hydrated tissue (RLWC^100^),


$$\text{AV}/{\text{V =(RLWC}}^{100}-\text{RLWC})/100$$

$${\text{RLWC}}^{100}-\text{RLWC})/100$$

The leaf electrolyte leakage (%) was determined as described by Mena-Petite et al. (2001). Three discs of 0.8 cm diameter from the full-expanded leaf´s left lateral leaflet were introduced into plastic vials, (previously washed with abundant deionized water) containing 16 mL of deionized water of known electrical conductivity (EC_0_). Electrolytic conductivity was measured using a digital conductivity meter (EC meter 19101-10 Cole-Parmer Intrument Company Vernon Hills, USA). The vial was closed and shaken to ensure that the plant material was in contact with the water. After 24 h at room temperature, a new electrical conductivity measurement was taken (EC_24_). Then, the samples were autoclaved (10 min 110°C), to disrupt the cell membranes and release all electrolytes from the cell and cooled at room temperature to measure the total electrical conductivity (EC_T_). The electrolyte leakage (EL, %), was calculated according the following equation:


$$\text{EL }\left(\%\right)=100\left(\;\frac{{\text{EC}}_{24}-{\text{EC}}_{0}}{{\text{EC}}_{\text{T}}-{\text{EC}}_{0}}\right)$$

### 2.6 Photosynthetic parameters

The determination of gas exchange parameters such as assimilation rate (μmol CO_2_·m^-2^·s^-1^) and stomatal conductance (gs) was performed the day before harvest using an infrared gas analyzer system (IRGA, CIRAS-2, PP Systems, Hitchin, UK) at a photon flux density of 700 μmol m^-2^ s^-1^. Measurements were taken in the terminal leaflet of the full-expanded leaf, 2 h after the lights of the chamber were switch on, with a cuvette at a stable temperature of 24°C, relative humidity of 60%, and the CO_2_ concentration at 410 ppm.

The photochemical efficiency of PSII in dark-adapted leaves, or also called maximum efficiency of PSII photochemistry, Fv/Fm (Genty et al., 1989), was measured in the central zone of the terminal leaflet of the full-expanded leaf the day before harvesting with a portable fluorometer (FluorPen FP 100, Photon Systems Instruments, spol. s.r.o., Drasov, Czech Republic). The Fv/Fm data in the plant adapted to dark conditions were taken in predawn conditions, while the Fv’/Fm’ data, were taken two hours after sunrise, once the plants were adapted to light conditions.

The determination of chlorophyll content was carried out by a portable chlorophyll meter (SPAD-502 plus, Konica Minolta Sensing, Inc), by measuring the terminal leaflet of the full-expanded leaf, the day before harvest (Adamsen et al., 1999).

### 2.7 ^13^C and ^15^N stable isotope analysis and C and N content

^13^C and ^15^N stable isotope analysis were performed as an estimation of WUE and biological nitrogen fixation, respectively. The determination of the isotopic ratio of ^15^N (∂^15^N) and the carbon isotope discrimination (Δ^13^C) was carried out from a pool of dry foliar material of each plant at the Research Support Service (SAI) of the Elvina Campus (A Coruña). Samples were ground and weighed into tin capsules using a microbalance UMX-2 balance (Mettler, Toledo). The combustion was performed on an elemental analyzer (Flash EA 1112, Thermo Finnigan) coupled *via* a ConfloII interface to an isotope ratio mass spectrometer (DeltaV Advantage, Thermo Scientific). The results for leaf C and N content were expressed in %, and those for ∂^13^C and ∂^15^N were expressed in ‰ relative to VPDB standards (Vienna Pee Dee Belemmite) and atmospheric air, respectively. The ratio (R) of ^13^C/^12^C was showed as δ^13^C (‰) indicating the C isotope composition:


$$\delta^{13}\text{C}=\left(\frac{{\text{R}}_{\text{sample}}}{{\text{R}}_{\text{standard}}}\right)-1$$

where, R_sample_ and R_standard_ are the isotope ratios (^13^C/^12^C) of the sample and international secondary standards of Known ^13^C/^12^C ratios (USGS 40, USGS41a, IAEA-N-1, IAEA-N-2 y USGS-25), respectively.

The natural ^15^N isotopic ratio (δ^15^N) was calculated using the formula described by Shearer et al. (1982):


$${\text{δ}}^{15}\text{N}=\frac{{\text{R}}_{\text{sample}}}{\left({\text{R}}_{\text{air}}-1\right)}*1,000$$

where, R_sample_ and R_air_ are the isotope ratios (^15^N/^14^N) of the sample and air, respectively.

The analysis of carbon isotope discrimination, Δ^13^C (‰) was calculated from the ∂^13^C (‰) values, according to, Polania et al. (2016),


$$\Delta^{13}C=\left(\frac{{\text{C}}_{\text{atm}}-\partial^{13}{\text{C}}_{\text{sample}}}{1}\right)+\frac{\partial_{sample}^{13}}{1,000}$$

where ∂^13^C_atm_ is the carbon isotope composition of atmospheric CO_2_ (−8‰) (Farquhar et al., 1989) and ∂^13^C_sample_ is the C isotope composition in seed samples.

From the values of leaf nitrogen content (LNC, %), the parameters of leaf nitrogen total content per plant (LNTC, g) was calculated.


LNTC=LNC∗leaf DW

where, DW was the total foliar biomass (g).

### 2.8 Soluble proteins

The extract used for the quantification of soluble proteins was carried out as Pérez-López et al. (2009b). The extracts were obtained using 0.15 g of fresh material from the central area of the full-expanded leaf collected at harvest and ground with liquid nitrogen on a previously cooled mortar. Eight plants per treatment were analyzed. The extract was homogenized with 3 mL of extraction buffer (50 mM phosphate buffer, pH 7.8; 0.1 mM EDTA; 5 mM Cysteine; 2 mM Asa), filtered through two layers of muslin and centrifuged at 4°C for 25 minutes at 16,100 *g*.

Five μl of supernatant were diluted in 595 μl of commercial Bradford reagent (BIO-RAD protein Assay) diluted in 1:4 MilliQ water. The reaction was maintained for 10 minutes in the darkness and the absorbance was determined at 595 nm (Bradford, 1976). A calibration curve was made with bovine serum albumin (BSA) (Abs_595_ = 0.34 mg prot · mL^-1^ + 0.0326; R^2^ = 0.990; using 8 calibration points, from 0 to 0.7 mg·mL^-1^ BSA to further determine the leaf protein concentration (LPC, mg prot g^-1^ DW). The leaf protein total content (LPTC, mg) was calculated from the LPC values, considering the foliar biomass (DW, g).


LPTC=LPC∗leaf DW

### 2.9 Statistical analysis

The data were analyzed using the statistical package SPSS Statistics 24.0 (IBM Corporation, Armonk, NY, USA). The normality of the non-standardized residuals of the data was studied using the Shapiro-Wilk test (considering normal data if p >0.05) and the homoscedasticity of the variance was studied with the Levenne test (considering homoscedasticity of the variance if p > 0.05). A two-way analysis of variance (ANOVA) was performed with water stress treatment and inoculation treatment as main factors and replicates as random effect. When the interaction between water treatment and inoculation treatment was significant, least square means *post-hoc* test was performed to compare means (Tukey or Kruskal Wallis, for parametric and non-parametric data, respectively).

To study the effect of the inoculation without presence of interaction, the multiple comparisons abovementioned were made comparing all the inoculation treatments with each other, but separating the data according to the water regime (capital letters for control conditions and lower case for drought conditions). The effect of water availability without presence of interaction was studied for each inoculation treatment separately performing a 1-factor ANOVA test, in the case of parametric data, or the U Mann-Withney test for two independent samples for non-parametric data indicating with asterisks the existence of significant differences.

For the correlations, the Pearson’s Coefficient for parametric data and Spearman’s for non-parametric data has been shown, also indicating the significance of these correlations by asterisks (* p< 0.05; ** p<0.01 and *** p<0.001).

In addition, a comparision between inoculated and non-inoculated plants was performed to evaluate the differences between plant with a non-inoculated treatment watered with mineral N-fertilization and plant inoculated with potentially drought tolerant rhizobia. Thus, data were analyzed using a two-way analysis of variance (ANOVA), with water stress treatment and “nitrogen source” category as main factor and replicates as random effect.

## 3 Results

### 3.1 Phenology and growth parameters

**Figure 1:**
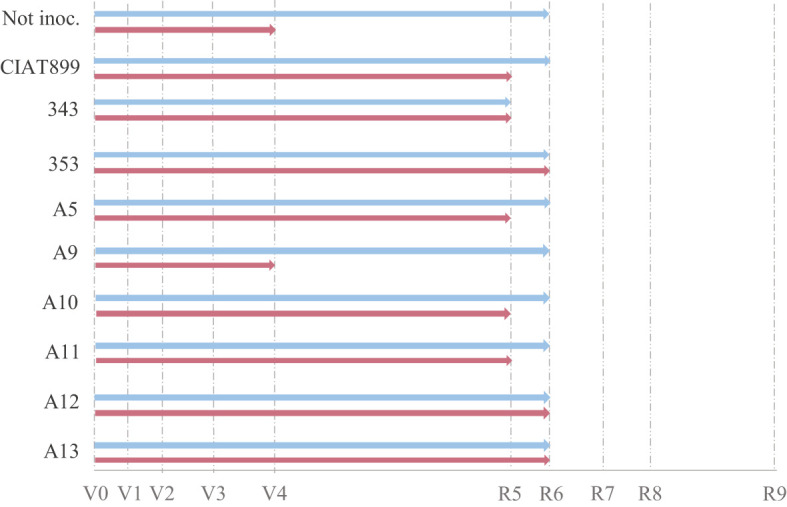
Phenological state of non-inoculated common bean (Not inoc.) and inoculated with different strains of rhizobium (CIAT899, 343, 353, A5, A9, A10, A11, A12 and A13), under different water availability conditions: well-watered (blue) and drought (red).

**Figure 2:**
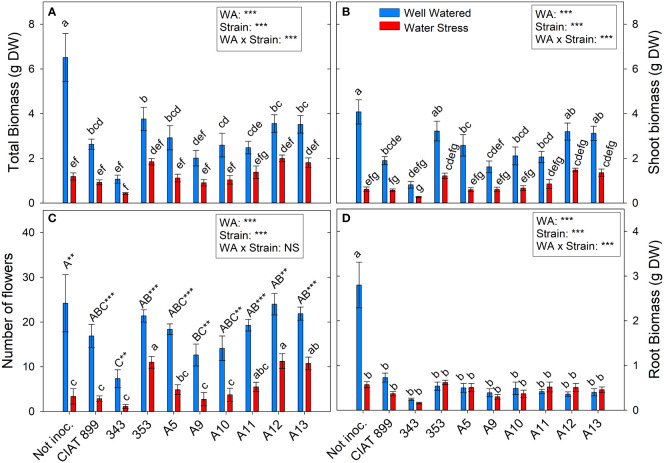
Phenological and biomass parameters of common bean plants without inoculum and nitrate-fertilized (not inoc.), and inoculated with different strains of rhizobium (CIAT899, A5, A9, A10, A11, A12 and A13), under different water availability (WA) conditions: well-watered (blue) and drought (red). When there is no interaction of factors, the multiple comparisons were made comparing all the inoculation treatments with each other, but separating the data according to the water regime (capital letters for control conditions and lower case for drought conditions), and the effect of water availability was studied for each inoculation treatment separately (using asterisks to show the effect): ** p<0.01 and *** p<0.001). **(A)** Total biomass, DW g; **(B)** Shoot biomass, DW g; **(C)** Number of flowers; and **(D)** Root biomass, DW (g) DW, dry weight.

**Figure 3:**
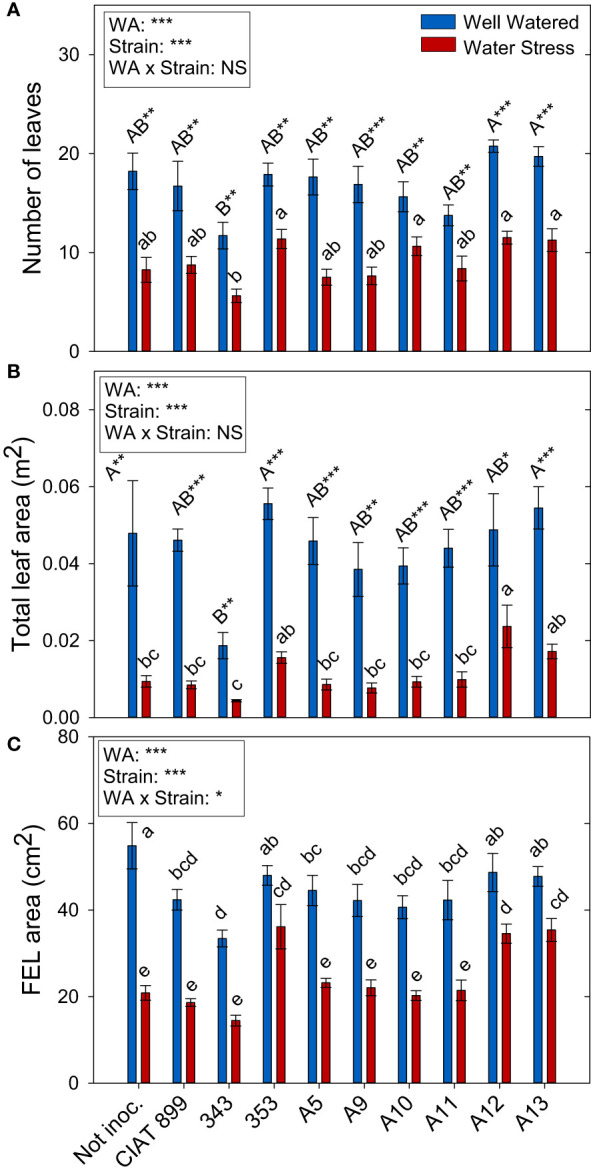
Leaf growth parameters of common bean plants without inoculum and nitrate-fertilized (not inoc.), and inoculated with different strains of rhizobium (CIAT899, A5, A9, A10, A11, A12 and A13), under different water availability (WA) conditions: well-watered (blue) and drought (red). When there is no interaction of factors, the multiple comparisons were made comparing all the inoculation treatments with each other, but separating the data according to the water regime (capital letters for control conditions and lower case for drought conditions), and the effect of water availability was studied for each inoculation treatment separately (using asterisks to show the effect): * p< 0.05; ** p<0.01 and *** p<0.001; NS, non-significant). **(A)** Number of leaves; **(B)** Total leaf area, m2; and **(C)** Full-expanded leaf (FEL) area, cm2.

Harvesting was done at flowering (R6) and all the studied phenological and growth parameters were affected both by the availability of water and by the inoculated strain (**Figures 1**–**3**). In general, drought delayed the development process by, at least, one stage (R5, **Figure 1** and **Supplementary Material Table S.1**) except in plants inoculated with the strains 353, A12 and A13 that showed the same developmental stage (R6) than well-watered plants at harvest (**Figure 1**). Plants inoculated with 353, A12 and A13 showed better response than the plants inoculated with the CIAT899 and especially those not inoculated (N-fertilized) that showed a significant phenological delay at harvest (vegetative state V4).

The number of flowers per plant (**Figure 2C**) was reduced from around 18 flowers in the well-watered conditions to 6 flowers under drought, a reduction of 65.95%. In well-watered conditions, no significant differences in the number of flowers were observed among the majority of inoculation treatments while, under drought, all inoculation treatments reduced significantly the number of flowers. However, with 353, A12 and A13 the reduction in flower number was lower than observed in the rest of treatments. Drought reduced on average for all treatment shoot biomass by 66.5% and total biomass by 59%. Non-inoculated plants (N-fertilized) (**Figure 2A**) produced the highest total biomass in well-watered conditions, especially in root biomass (**Figure 2D**), but were the treatment most negatively affected by drought. In inoculated plants, drought reduced all biomass parameters except for the root biomass (**Figure 2D**), and no differences among strains were observed, although the plants inoculated with the strain 353, A12 and A13 reached the highest values of leaf biomass under water deficit conditions, surpassing N-fertilized and plants inoculated with strain CIAT 899 (**Supplementary Figure 1**).

Total leaf area (**Figure 3B**) was more negatively affected by drought than leaf biomass (**Supplementary Figure 1**). This fall in total leaf area was due to a reduction in the number of leaves per plant (46%, **Figure 3A**) and a reduction of the size of the leaves (full-expanded leaf, 43.9%, **Figure 3C**). In general, in well-watered conditions, plants not inoculated and those inoculated with the 353, A12 and A13 strains showed the highest values in biomass parameters whereas, under drought, these strains responded better than the nitrogen fertilized plants and the plants inoculated with CIAT 899 (**Figure 3**). In the other hand, the strain 343 showed the lowest value both in control and drought conditions in all biomass parameters evaluated.

### 3.2 Water relations

**Figure 4:**
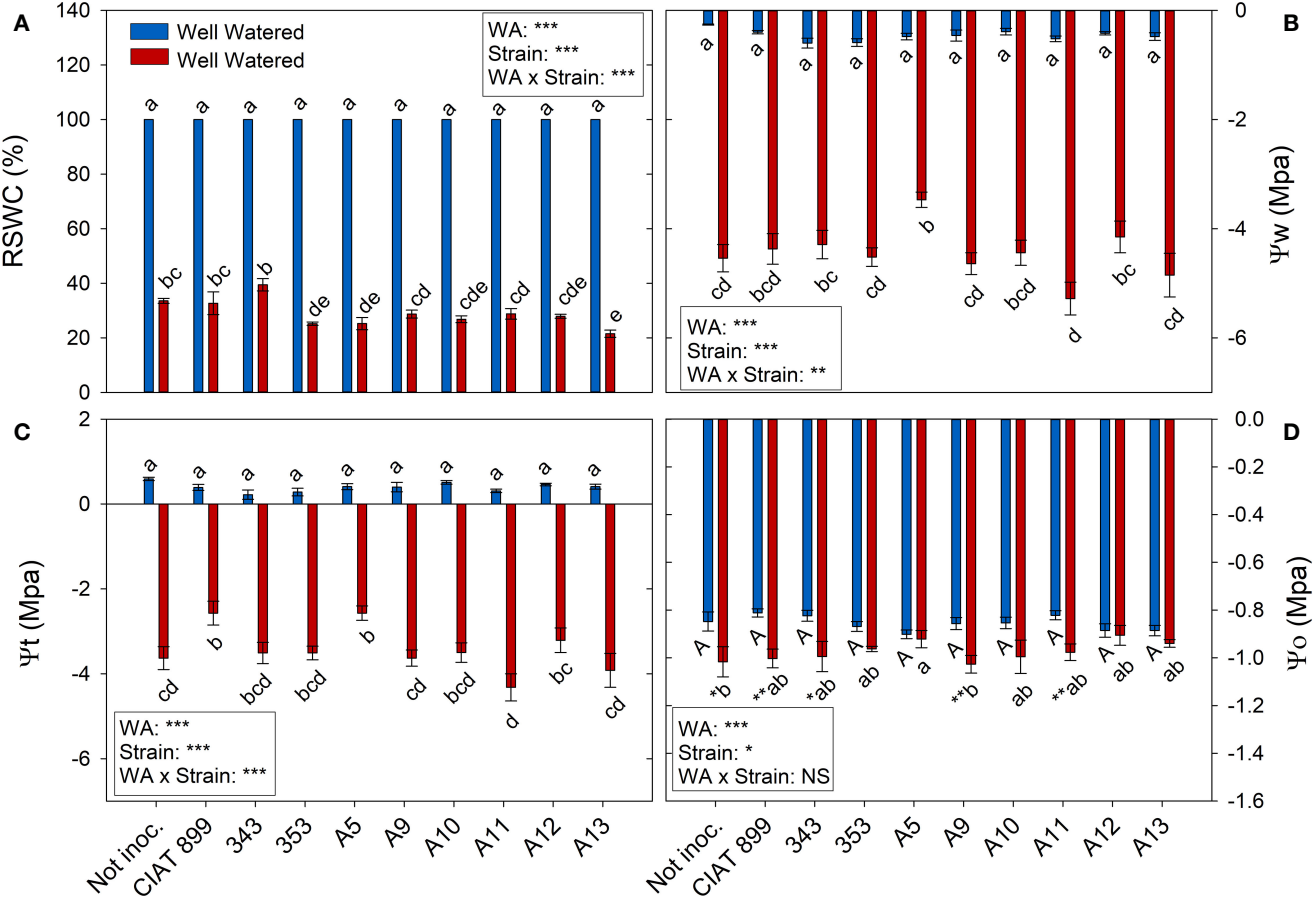
Relative soil water content (RSWC) and water potential parameters of common bean plants without inoculum and nitrate-fertilized (not inoc.), and inoculated with different strains of rhizobium (CIAT899, A5, A9, A10, A11, A12 and A13), under different water availability (WA) conditions: well-watered (blue) and drought (red). When there is no interaction of factors, the multiple comparisons were made comparing all the inoculation treatments with each other, but separating the data according to the water regime (capital letters for control conditions and lower case for drought conditions), and the effect of water availability was studied for each inoculation treatment separately (using asterisks to show the effect): * p< 0.05; ** p<0.01 and *** p<0.001; NS, non-significant). **(A)** Relative soil water content, RSWC %; **(B)** Water potential, Ψw MPa); **(C)** Cell wall pressure potential, Ψt MPa); and **(D)** Osmotic potential, Ψo MPa.

**Figure 5:**
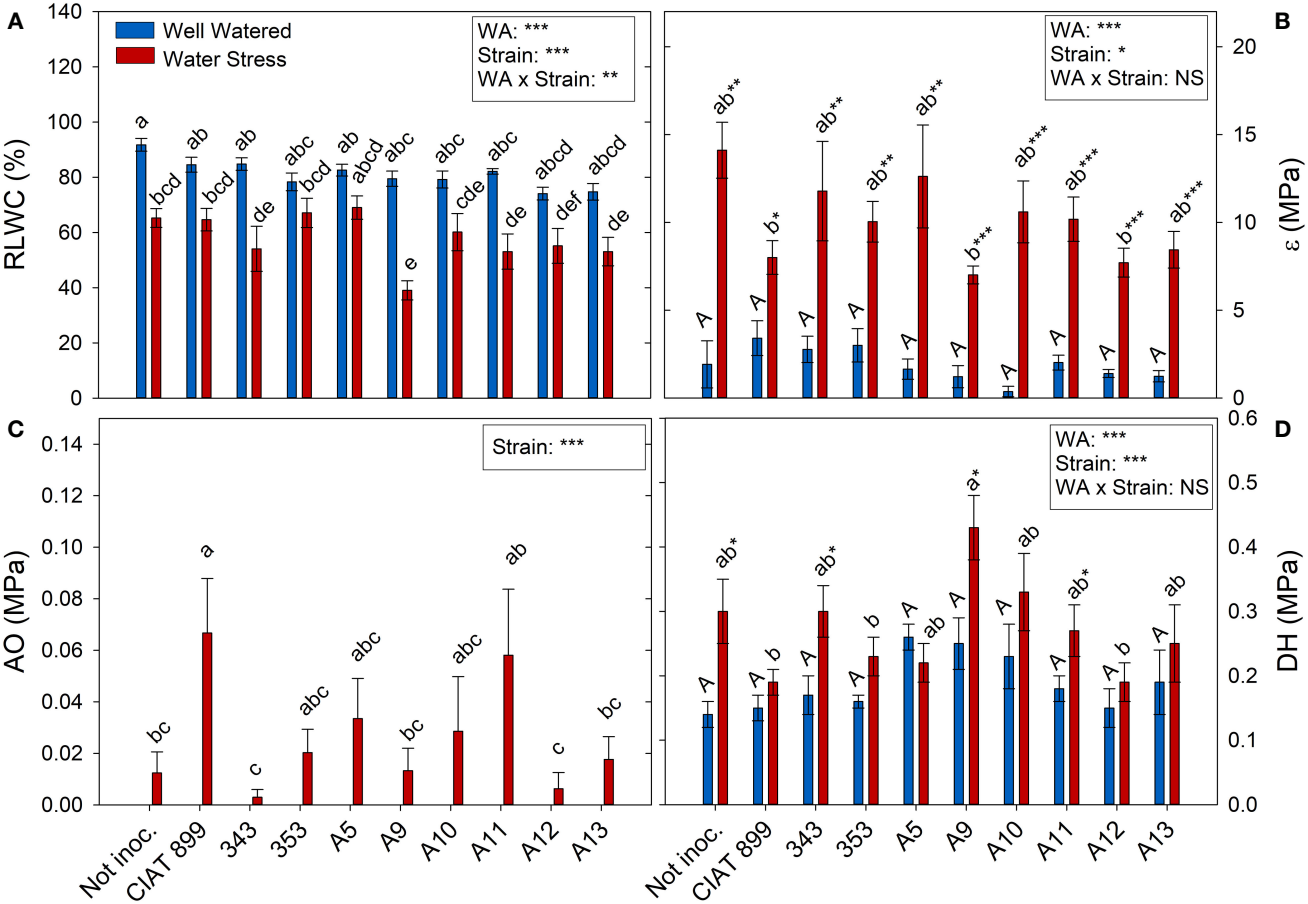
Relative leaf water content, **(A)** RLWC %; **(B)** Cell elastic modulus, ϵ MPa; **(C)** Osmotic adjustment, OA MPa; and **(D)** Dehydration, DH MPa, of common bean plants without inoculum and nitrate-fertilized (not inoc.), and inoculated with different strains of rhizobium (CIAT899, A5, A9, A10, A11, A12 and A13), under different water availability (WA) conditions: well-watered (blue) and drought (red). When there is no interaction of factors, the multiple comparisons were made comparing all the inoculation treatments with each other, but separating the data according to the water regime (capital letters for control conditions and lower case for drought conditions), and the effect of water availability was studied for each inoculation treatment separately (using asterisks to show the effect): * p< 0.05; ** p<0.01 and *** p<0.001; NS, non-significant).

At harvest, control plants were at 100% of field capacity while in drought stressed plants the relative water content of the soil was close to 30% (20% in plants inoculated with strain A13, **Figure 4A**), confirming the drought level at which different treatments were exposed. The drought treatment significantly decreased leaf water potential (Ψw), from an average value of -0.46 MPa in the well-watered control treatment, to an average value of -4.46 MPa under drought (**Figure 4B**). The inoculation treatment showed no differences in Ψw in within the well-watered plants although under drought, the plants inoculated with the strain A11 reached the lowest water potential values (-5.28 MPa) and those inoculated with A5 the highest one (-3.47 MPa). This drop in Ψw was mainly due to the loss of cellular turgor, due to a decrease in cell wall pressure potential (Ψt, **Figure 4C**) showing a very similar behaviour in most inoculated plants. In the other hand, in the non-inoculated plants and in the inoculated plants with CIAT899, 343, A9 and A11, the drop in Ψw was also due to a significant drop in osmotic potential (Ψo, **Figure 4D**). This drop in Ψo could be due to a passive loss of water, dehydration (**Figure 5D**), as observed in non-inoculated and in plants inoculated with 343, A9 and A11 strains that showed also a reduction in the relative water content of the leaf (**Figure 5A**). This decrease could also be due to an active process of solutes accumulation (osmotic adjustment, AO **Figure 5C**) being the plants inoculated with reference strain CIAT899 and A11 the ones that showed the highest AO values while the inoculated with 343 showed the lowest.

**Figure 6:**
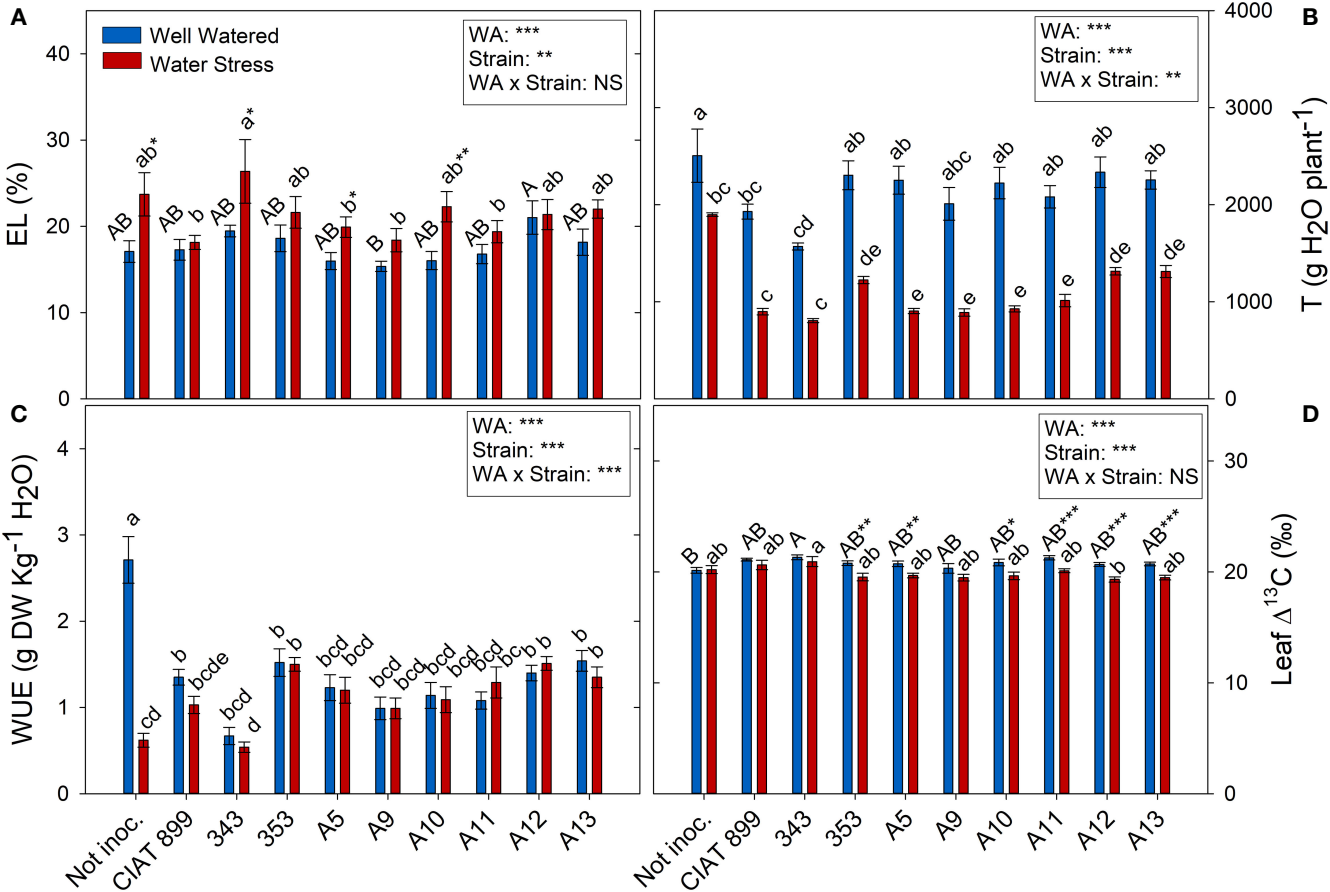
Electrolyte leakage and transpiration related parameters of common bean plants without inoculum and nitrate-fertilized (not inoc.), and inoculated with different strains of rhizobium (CIAT899, A5, A9, A10, A11, A12 and A13), under different water availability (WA) conditions: well-watered (blue) and drought (red). When there is no interaction of factors, the multiple comparisons were made comparing all the inoculation treatments with each other, but separating the data according to the water regime (capital letters for control conditions and lower case for drought conditions), and the effect of water availability was studied for each inoculation treatment separately (using asterisks to show the effect): * p< 0.05; ** p<0.01 and *** p<0.001; NS, non-significant). **(A)** Electrolyte leakage, EL %; **(B)** Accumulated transpiration, T g H2O·plant-1; **(C)** Water use efficiency, WUE g DW·Kg-1 H2O; and **(D)** Leaf Δ13C, ‰. DW, dry weight.

All the plants showed a significant increase in the elasticity of the cell wall (ϵ) with drought stress (**Figure 5B**) being significantly lower in plants inoculated with bacteria, especially in plants inoculated with A9, A12, CIAT899, A13 and 353 strains. Drought also caused a degradation of the membranes as indicated by the increase in electrolyte leakage (**Figure 6A**), being the plants inoculated with A12 and the CIAT899 those least affected by membrane leakage; whereas the highest values were observed in not inoculated plants and those inoculated with strains 343 and A10, the most drought sensitive inoculation treatments.

Concerning the total transpiration accumulated during the whole experiment, non-inoculated plants were the ones that showed highest values under well-watered conditions and especially under drought (**Figure 6B**), therefore, they showed a large drop in the total water use efficiency (WUE) under drought (76.9%, **Figure 6C**). Among inoculated plants, drought reduced total transpiration and got similar WUE than control plants. The lowest WUE was observed in plants inoculated with 343 strains whereas plants inoculated with strains 353, A12 and A13 showed higher WUE values and lower carbon isotope discrimination (Δ^13^C) values (**Figure 6D**) under drought.

### 3.3 Photosynthetic parameters

**Figure 7:**
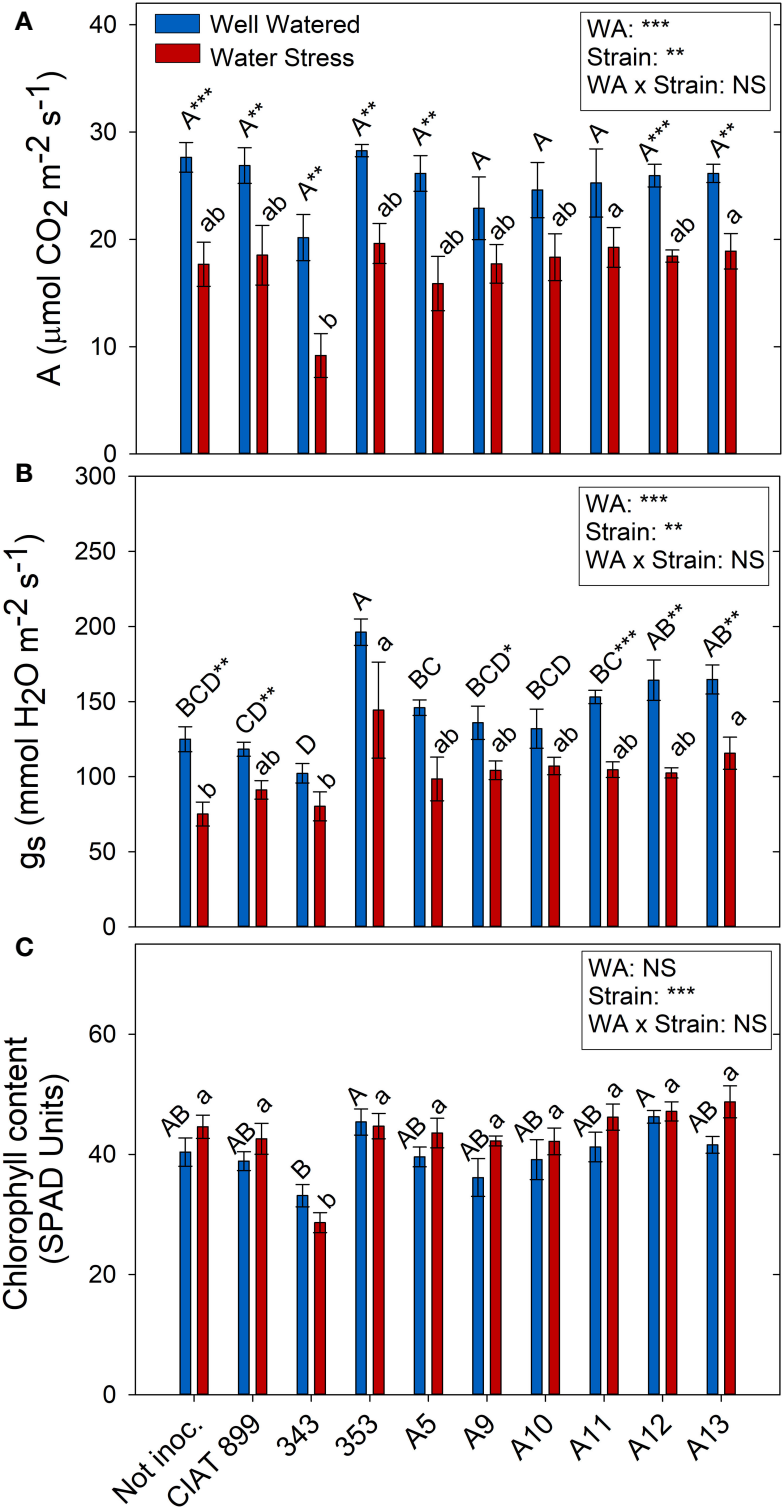
Photosynthetic parameters of common bean plants without inoculum and nitrate-fertilized (not inoc.), and inoculated with different strains of rhizobium (CIAT899, A5, A9, A10, A11, A12 and A13), under different water availability (WA) conditions: well-watered (blue) and drought (red). When there is no interaction of factors, the multiple comparisons were made comparing all the inoculation treatments with each other, but separating the data according to the water regime (capital letters for control conditions and lower case for drought conditions), and the effect of water availability was studied for each inoculation treatment separately (using asterisks to show the effect): * p< 0.05; ** p<0.01 and *** p<0.001; NS, non-significant). **(A)** Assimilation rate, A μmol CO2·m-2·s-1); **(B)** Stomatal conductance, mmol H2O·m-2·s-1; **(C)** Chlorophyll content, SPAD units.

Drought and the different strains had a direct effect on photosynthesis (A) and stomatal conductance (gs) but no interaction between these factors two factors was found (**Figure 7**). Drought reduced on average for all treatments photosynthesis by 32%, and stomatal conductance by 30%. However, photochemical efficiency of PSII in dark adapted plants (Fv/Fm) were not affected by drought and all values were close to 0.8 (data not shown). Similar response was detected in light-adapted leaves (Fv´/Fm´) that were not affected neither by drought nor by inoculation treatments. Similarly, no significant differences were observed in the chlorophyll content among treatments, except for plants inoculated with the 343 strain under drought conditions (**Figure 7C**).

The leaf C% was affected by water deficit in the plants inoculated with 343, A9 and A11 strains (supplementary material **Supplementary Figure 2A**), while the plants inoculated with the strains CIAT899 and A5 showed the highest percentage under both water availability treatments. The total leaf carbon content (mg) was higher in plants inoculated with the strains 353, A12 and A13 both in well-watered conditions and under drought, whereas the lowest values were obtained in the plants inoculated with the strain 343 (supplementary material **Supplementary Figure 2B**).

### 3.4 Nitrogen fixation and content

**Figure 8:**
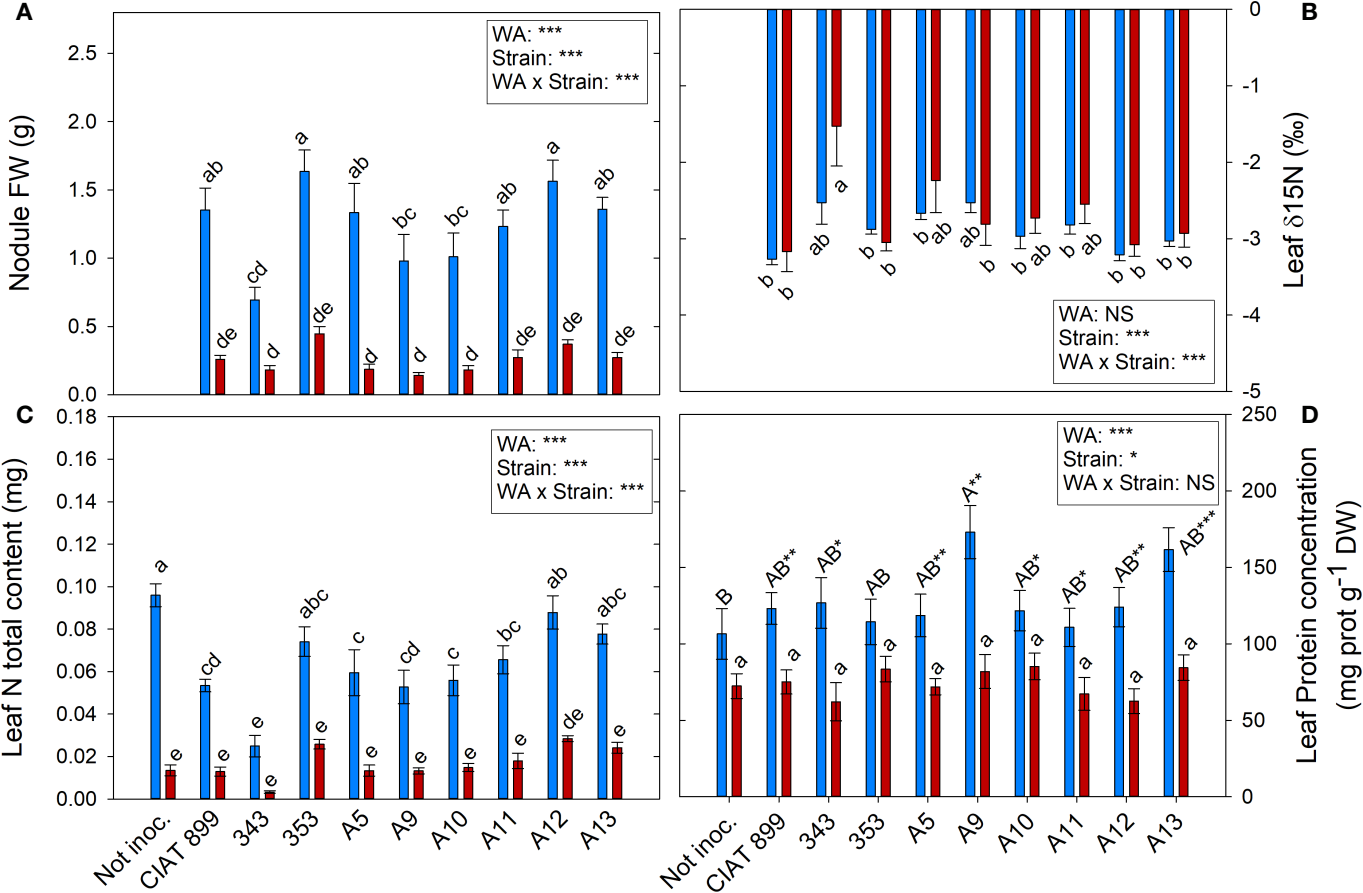
Nitrogen fixation related parameters of common bean plants without inoculum and nitrate-fertilized (not inoc.), and inoculated with different strains of rhizobium (CIAT899, A5, A9, A10, A11, A12 and A13), under different water availability (WA) conditions: well-watered (blue) and drought (red). When there is no interaction of factors, the multiple comparisons were made comparing all the inoculation treatments with each other, but separating the data according to the water regime (capital letters for control conditions and lower case for drought conditions), and the effect of water availability was studied for each inoculation treatment separately (using asterisks to show the effect): * p< 0.05; ** p<0.01 and *** p<0.001; NS, non-significant). **(A)** Nodule fresh weigh, g; **(B)** leaf δ15N, %; **(C)** leaf N total content, mg; **(D)** leaf protein concentration, %.

The highest nodule production was observed in plants inoculated with 353 and A12 strains in well-watered conditions (1.63 and 1.56 g per plant, respectively) and were also the ones that showed the highest nodule biomass (0.447 and 0.371 g respectively) under drought conditions although the stress reduced by more than 70% the nodule biomass (**Figure 8A**). The strain with the lowest δ^15^N values, which represents higher biological nitrogen fixation, were CIAT899, 353, A12 and A13 in both water availably treatments while 343 presented the lowest, especially under drought (**Figure 8B**).

Drought reduced the overall N% content of leaves by 26.99%, being the decrease significant in the plants inoculated with the strains CIAT899, A11, A13 and especially in 343 (**Supplementary Figure 3A**). The higher leaf total N content under well-watered conditions was found in plants fertilized with N and those inoculated with the strain 353, A12 and A13 (**Figure 8C**). Drought reduced this value in all inoculation treatments being the plants inoculated with A12 the ones that showed lower reduction.

### 3.5 Soluble protein

The foliar concentration of soluble proteins (**Figure 8D**) and total leaf protein content (supplementary material **Supplementary Figure 3B**) were affected by water supply and inoculation treatments. Under irrigation conditions, non-inoculated plants showed the lowest leaf protein concentration in comparison with rhizobia inoculation treatments, reaching the maximum value in plants inoculated with strain A9 and A13. Protein content decreased an average of 40.87% under drought conditions, however, no significant differences were observed among inoculation treatments. The total leaf protein content showed a similar behaviour to the observed in the total N content of the foliar biomass (**Figure 8B**).

### 3.6 Correlations between studied parameters

A strong positive correlation was detected between the developmental stage and the number of flowers per plant at harvest time (R=0.848***, **Table 2**); as well as between the number of flowers and both shoot and foliar biomass (R=0.833*** and R=0.825*** respectively), and between the number of flowers and nodule fresh weight (R=0.704***). A weaker correlation, although significant, was observed between the developmental stage and shoot biomass (R=0.647***) and between the number of flowers and the assimilation rate (R=0.638***, **Table 2**). The correlations between number of flowers and developmental stage with the total biomass was also significant, R=0.791*** and R=0.582***, respectively (**Table 2**).

**Table 2:** Pearson’s correlation indices (R) between the analyzed parameters: Transpiration (T, g H_2_O·plant-1), water use efficiency (WUE, g DW·Kg-1H_2_O), relative leaf water content (RLWC, %), water potential (Ψw, MPa), osmotic potential (Ψo, MPa), cell wall pressure potential (Ψt, MPa), dehydration (DH, MPA), osmotic adjustment (OA, MPa), electrolyte leakage (EL, %), nodule fresh weigh (NFW, g), flower number (FN), developmental stage (DS), total leaf area (TLA, m2), root biomass (RB, g), shoot biomass (SB, g), total biomass (TB, g), isotopic ratio of ^15^N (δ^15^N, ‰), leaf N concentration (N, %), assimilation rate (A, µmol CO2·m-2·s-1) and cell elastic modulus (ε, MPa) (indicating the significance of these correlations by asterisks (* p < 0.05 and ** p <0.01).

	T	WUE	RLWC	Ψw	Ψo	Ψt	DH	OA	EL	NFW	FN	DS	TLA	RB	SB	TB	δN^15^	N%	A	ϵ
**T**	1	,448^**^	,508^**^	,757^**^	,226^**^	,752^**^	-,252^**^	-,357^**^	-,263^**^	,731^**^	,770^**^	,616^**^	,794^**^	,442^**^	,855^**^	,797^**^	0,096	,347^**^	,642^**^	-,604**
**WUE**	,448^**^	1	0,079	,228^**^	0,023	,240^**^	-,168^*^	-0,054	-0,084	,189^*^	,596^**^	,481^**^	,487^**^	,779^**^	,751^**^	,838^**^	,200^*^	-0,067	,378^**^	-,286**
**RLWC**	,508^**^	0,079	1	,655^**^	,320^**^	,663^**^	-,286^**^	-,278^**^	-,341^**^	,411^**^	,402^**^	,300^**^	,445^**^	0,139	,351^**^	,313^**^	,191^*^	,548^**^	,313^**^	-,243**
**Ψw**	,757^**^	,228^**^	,655^**^	1	,440^**^	,987^**^	-,309^**^	-,387^**^	-,398^**^	,690^**^	,667^**^	,570^**^	,722^**^	,191^*^	,623^**^	,539^**^	0,055	,611^**^	,550^**^	-,728**
**Ψo**	,226^**^	0,023	,320^**^	,440^**^	1	,408^**^	-,489^**^	-,217^**^	-,184^*^	,248^**^	,201^*^	,232^**^	,306^**^	0,054	,159^*^	0,140	0,119	,309^**^	,171^*^	-,353**
**Ψt**	,752^**^	,240^**^	,663^**^	,987^**^	,408^**^	1	-,315^**^	-,350^**^	-,403^**^	,696^**^	,658^**^	,555^**^	,722^**^	,196^*^	,630^**^	,547^**^	0,040	,613^**^	,584^**^	-,741**
**DH**	-,252^**^	-,168^*^	-,286^**^	-,309^**^	-,489^**^	-,315^**^	1	-0,132	,209^*^	-,246^**^	-,227^**^	-,218^*^	-,294^**^	-0,165	-,273^**^	-,262^**^	-0,103	-,241^**^	-,199^*^	,172*
**OA**	-,357^**^	-0,054	-,278^**^	-,387^**^	-,217^**^	-,350^**^	-0,132	1	-0,058	-,263^**^	-,307^**^	-,262^**^	-,291^**^	-0,057	-,265^**^	-,221^**^	-0,056	-0,155	-,168^*^	,302**
**EL**	-,263^**^	-0,084	-,341^**^	-,398^**^	-,184^*^	-,403^**^	,209^*^	-0,058	1	-,239^**^	-,172^*^	-0,122	-,327^**^	-0,076	-,218^**^	-,187^*^	0,035	-,442^**^	-,177^*^	,237**
**NFW**	,731^**^	,189^*^	,411^**^	,690^**^	,248^**^	,696^**^	-,246^**^	-,263^**^	-,239^**^	1	,704^**^	,596^**^	,676^**^	-0,058	,647^**^	,472^**^	-,366^**^	,333^**^	,553^**^	-,576**
**FN**	,770^**^	,596^**^	,402^**^	,667^**^	,201^*^	,658^**^	-,227^**^	-,307^**^	-,172^*^	,704^**^	1	,848^**^	,775^**^	,373^**^	,833^**^	,791^**^	-0,077	,292^**^	,638^**^	-,580**
**DS**	,616^**^	,481^**^	,300^**^	,570^**^	,232^**^	,555^**^	-,218^*^	-,262^**^	-0,122	,596^**^	,848^**^	1	,632^**^	,254^**^	,647^**^	,582^**^	-,184^*^	,227^**^	,534^**^	-,539**
**TLA**	,794^**^	,487^**^	,445^**^	,722^**^	,306^**^	,722^**^	-,294^**^	-,291^**^	-,327^**^	,676^**^	,775^**^	,632^**^	1	,366^**^	,784^**^	,724^**^	0,004	,349^**^	,679^**^	-,590**
**RB**	,442^**^	,779^**^	0,139	,191^*^	0,054	,196^*^	-0,165	-0,057	-0,076	-0,058	,373^**^	,254^**^	,366^**^	1	,580^**^	,782^**^	,523^**^	-0,044	,270^**^	-,172*
**SB**	,855^**^	,751^**^	,351^**^	,623^**^	,159^*^	,630^**^	-,273^**^	-,265^**^	-,218^**^	,647^**^	,833^**^	,647^**^	,784^**^	,580^**^	1	,961^**^	0,114	0,140	,610^**^	-,541**
**TB**	,797^**^	,838^**^	,313^**^	,539^**^	0,140	,547^**^	-,262^**^	-,221^**^	-,187^*^	,472^**^	,791^**^	,582^**^	,724^**^	,782^**^	,961^**^	1	,258^**^	0,092	,558^**^	-,469**
**δN^15^**	0,096	,200^*^	,191^*^	0,055	0,119	0,040	-0,103	-0,056	0,035	-,366^**^	-0,077	-,184^*^	0,004	,523^**^	0,114	,258^**^	1	-0,014	-0,073	0,118
**N%**	,347^**^	-0,067	,548^**^	,611^**^	,309^**^	,613^**^	-,241^**^	-0,155	-,442^**^	,333^**^	,292^**^	,227^**^	,349^**^	-0,044	0,140	0,092	-0,014	1	,298^**^	-,427**
**A**	,642^**^	,378^**^	,313^**^	,550^**^	,171^*^	,584^**^	-,199^*^	-,168^*^	-,177^*^	,553^**^	,638^**^	,534^**^	,679^**^	,270^**^	,610^**^	,558^**^	-0,073	,298^**^	1	-,508**
**ϵ**	-,604**	-,286**	-,243**	-,728**	-,353**	-,741**	,172*	,302**	,237**	-,576**	-,580**	-,539**	-,590**	-,172*	-,541**	-,469**	0,118	-,427**	-,508**	1

Among the water relation parameters, the accumulated transpiration depends on the shoot biomass and total leaf area of the plants as shown by the strong correlation observed between both parameters, R=0.855*** and R=0.794***, respectively (**Table 2**). A strong correlation was also obtained between Ψw and Ψt (R=0.987***), compared to the weaker correlation between Ψw and Ψo (R=0.440***, **Table 2**). A positive correlation was also detected between the Ψt and the shoot biomass (R=0.630***, **Table 2**). The correlations obtained between Ψo and osmotic adjustment (OA) and dehydration (DH) confirms that in the case of CIAT899 the drop in osmotic potential was associated with the osmotic adjustment (**Table 3**). In the case of plants inoculated with the strains 353 and A11, the drop in Ψo was correlated both to OA and to dehydration. By the contrary, in non-inoculated plants and plants inoculated with 343, A9 and A10 strains, the drop in Ψo was correlated to dehydration. A positive correlation was also detected between ϵ values and electrolyte leakage (R=0.237**).

**Table 3:** Pearson’s correlation indices (R) between Ψo and the parameters of osmotic adjustment (OA) and dehydration (DH), in common bean plants without microsymbiont (not inoc.) and inoculated with different strains of rhizobium: CIAT 899, A5, A9, A10, A11, A12 and A13 (* p< 0.05; ** and p<0.01).

	DH	OA
Not Inoc.	-0.696**	-0.198
CIAT899	-0.469	-0.692*
343	-0.681*	0.12
353	-0.757*	-0.657*
A5	-0.309	-0.036
A9	-0.75**	-0.186
A10	-0.603*	0.134
A11	-0.677**	-0.577*
A12	-0.365	-0.208
A13	-0.205	-0.065

The relationship of CO_2_ assimilation rate (A) and biomass production occurred mainly between shoot biomass and assimilation rate, rather than between total biomass and A, as shown by the correlation coefficients obtained (R=0.610 ** and R=0.558 ** respectively). A correlation between total leaf area and assimilation rate (R=0.679 ***, **Table 2**), and between the relative leaf water content and assimilation rate (R=0.313***, **Table 2**) were also detected

A positive correlation was observed between nodule fresh weight per plant and shoot biomass production (R=0.647 ***), and negative between nodule fresh weight and isotopic analysis of ^15^N (R=-0.366 **, **Table 2**).

## 4 Discussion

### 4.1 Water relations

Common bean plants are considered to be water stressed when soil reaches 50% relative water content (Sánchez-Reinoso et al., 2019; Nawaz et al., 2021). The plants in this trial were exposed to values of 30% relative soil water content at the time of harvest, which is considered a severe drought (Xu and Zhou, 2006). This affected considerably the growth and development of the plants with a reduction of 66.5% in shoot biomass, 74.1% in total leaf area, a decrease in number and size of leaves and in number of flowers. This soil water content, caused a large drop in the water potential, reaching an average value of -4.46 MPa, very low values compared to the reached in others water stress studies (-1.5 MPa) with common bean (Hageman et al., 2020). The drop in Ψw was mainly due to the drop in the pressure potential (Ψt), especially in the non-inoculated plants and in those inoculated with CIAT899, 343, A9 and A11 strains, a drop in osmotic potential was also significant.

Although a slight osmotic adjustment was detected mainly in plants inoculated with the reference strain CIAT 899, in the non-inoculated plants and in some of the more drought sensitive strains (especially 343); the drop in osmotic potential was due to dehydration. In addition, these plants suffered a great increase in the elasticity of the cell wall and a significant reduction in the relative water content of the leaves, as has also been reported in other not very successful bacterial inoculation tests under drought (Curá et al., 2017; Sapna and Sharma, 2020; Steiner et al., 2020). The cell wall elasticity adjustment is considered to be one of the most important physiological mechanisms of tolerance to water stress in several species (Marshall & Dumbroff, 1999; De Diego et al., 2013). This helps to maintain cellular turgor and volume and prevents mechanical damages in the plasma membrane, (Mena-Petite et al., 2001; De Diego et al., 2013) as indicated by low values of electrolyte leakage of the plants inoculated with CIAT899, A12, A13 and 353 strains. These plants also showed the lowest increase of ϵ values under drought, maintaining a better adjustment of the cell wall elasticity and higher leaf water content, as confirm the detected correlation between ϵ values and electrolyte leakage (R=0.237**). These results agree with many other studies that shown that inoculation with efficient strains reduces membrane damage in severe stress situations in different legumes such as soybean (Abbasi et al., 2013; Igiehon & Babalola, 2017; Kibido et al., 2019) or common bean (Steiner et al., 2020). This could explain the higher drought tolerance of CIAT899, 353, A12 and A13 strains which showed lower dehydration, higher leaf water content and, in the case of CIAT899, a better osmotic adjustment. In this sense, the accumulation of osmolytes such as trehalose and proline in the nodules has been related to the tolerance to drought of some plant genotypes (Goyal et al., 2021; Santillana Villanueva, 2021).

### 4.2 Photosynthetic parameters

Drought reduced the photosynthetic assimilation in most inoculation treatments due in part to stomatal limitations of photosynthesis (**Figure 6**) (Robredo et al., 2007) and did not produced any photochemical limitations since there were no significant changes in Fv/Fm nor in Fv’/Fm’ values. These results indicate the absence of damage at the PSII level and that the capture and transduction of energy from the antenna complex to the PSII was not affected (Mathobo et al., 2017; Calzone et al., 2020). In addition, similarly to the observations of Boydston et al. (2018), plants subjected to water deficit did not show significant differences in their chlorophyll content (SPAD values). Among all the plants, those inoculated with 353, A12 and A13 indigenous strains together with the reference strain CIAT899 showed the highest photosynthesis and the highest percentage of leaf C concentration under drought, contrary to the plants inoculated with the 343 strain. In addition, similarly to Sapna & Sharma (2020), a positive strong correlation was detected between photosynthetic rate and relative leaf water content and, according to their observations, both parameters are related to grain yield in Mungbean plants inoculated with drought efficient rhizobia strains, confirming the efficiency of the aforementioned strains (CIA899, 353, A12 and A13).

### 4.3 Water use efficiency

As consequence of stomatal closure and the reduction of number and biomass of leaves under drought, the whole plant transpiration was reduced to cope with lack of water, maintaining the whole plant water use efficiency similar to the control plants (WUE), that decreased only in non-inoculated plants. Although under drought conditions, plants generally tend to increase WUE (Polania et al., 2016; Sanz‐Saez et al., 2019) under severe stomatal closure, the efficiency of water use decreases again (Salazar-Tortosa et al., 2018). Thus, no differences were found in the WUE among the inoculation treatments. However, when considering the carbon isotope discrimination (Δ^13^C), another way of measuring the water use efficiency of plants since both parameters are negatively correlated (Farquhar et al., 1989), bigger differences were observed. Thus, strains 353, A12 and A13, showed higher water use eficiency (lower Δ^13^C) under drought, while the plants inoculated with CIAT899 did not showed any change. This confirmed the efficiency of these three strains (353, A12 and A13), since WUE or “more crop per drop” is considered as an important component of drought tolerance in different crops (Blum, 2009; Polania et al., 2016).

### 4.4 Nitrogen fixation and content

In inoculated plants the only source of nitrogen was the atmospheric N_2,_ accordingly, all the N content in these plants came from the biological nitrogen fixation (BFN); therefore, the total N content in the tissues is an estimate of the efficiency of the symbiosis. In addition, a positive correlation between the N% and nodule fresh weight was detected (R=0.333***). There are many studies using nodule weight as an indicator of a strain´s infectiveness and many others report that drought reduces nodulation (Kibido et al., 2019; Aulakh et al., 2020; Santillana Villanueva, 2021). It has also been stated that the more drought tolerant a strain is, the greater nodulation capacity it has under drought conditions (Aulakh et al., 2020; Steiner et al., 2020; Omari et al., 2022). The negative correlation between nodules fresh weight and ∂^15^N (R=-0.366**) would confirm that the greater the biomass of nodules, the greater the capacity for nitrogen fixation. From all the strains tested, plants inoculated with 353, A12, A13 and CIAT899 bacteria showed the highest infectiveness (higher nodule weight) and the lowest δ^15^N values (higher biological nitrogen fixation) in both water availably treatments. The most efficient strains allow plants to develop a greater number of nodules and fix more nitrogen under stress conditions, resulting in a greater accumulation of N in their tissues higher leaf chlorophyll content and more shoot biomass (Steiner et al., 2020; Rodiño et al., 2021; Omari et al., 2022), in a similar way to what was observed in plants inoculated with the strain 353, A12 and A13, confirming these strains as the ones with the highest nitrogen fixation, highlighting the strain A12. On the contrary, the strain 343 showed the worst behaviour with the lowest values in all parameters tested.

### 4.5 Phenology and growth parameters

Biomass production is also usually used as strain effectiveness indicator (Kibido et al., 2019; Aserse et al., 2020). In this study, there was a high correlation (R=0.647 ***) between nodule fresh weight and shoot biomass. Thus, the strains with the highest nodulation capacity under drought (353, A12, A13 and CIAT899) were those with the highest biomass production, except in the case of CIAT899, which did not develop much shoot biomass. On the contrary, the plants inoculated with 343 rhizobia, presented the lowest shoot dry weight in both water treatments, confirming the low efficiency of this strain. Furthermore, shoot dry weight has also been related to grain yield in several crop species such as common bean (Beebe et al., 2013; Rai et al., 2020). Therefore, the strains capable of increasing the shoot biomass are expected to achieve higher yields as confirmed in many studies (Igiehon et al., 2021), which is why it is considered a criterion for the selection of inoculants in addition to an indicator of strains effectiveness (Aserse et al., 2020; Aulakh et al., 2020). In our study this is further confirmed by the strong positive correlation between nodule fresh weight and number of flowers (R=0.704 ***). In fact, Omari et al. (2022) found that the number of nodules was positively correlated with soybean yield and protein content, confirming many previous reports that concluded that nodulation enhancement is essential for increasing soybean grain yield (Thilakarathna and Raizada, 2017; Moretti et al., 2018). Therefore, the strain 353, A12 and A13 were the most efficient strains under drought stress, for the studied genotype, surpassing even the reference strain CIAT899. These strains showed as well the highest total leaves area in both water availability conditions, keeping higher photosynthetic capacity (higher total content of C in leaves) per plant.

Drought delayed the phenological development of the plants by one stage (from R6 in well-watered conditions to R5) and reduced the number of flowers by 65.9% since drought stress affects flower initiation (Nuñez Barrios et al., 2005), as well as flower abortion (Monteiro-Galvao et al., 2019) affecting yield (Nuñez Barrios et al., 2005; Steiner et al., 2020). However, inoculation of Arrocina de Álava plants with efficient strains (353, A12 and A13) avoided this phenological delay keeping them in R6 like the well-watered plants and also developed a greater number of flowers, even improving the effects of the inoculation with CIAT899 reference strain. On the contrary, the nitrate fertilized plants showed the greatest delay in development under drought remaining in a vegetative state (V4). Thus, the inoculation of efficient strains increased the plant tolerance by accelerating flowering, in the studied genotype, which is considered a common strategy for “evasion” of water stress, since it allows plants to escape the drought by completing their life cycle before the onset of severe stress conditions (Polania et al., 2016; Sedlar et al., 2020).

### 4.6 Selection of tested drought tolerant indigenous rhizobia

The results of this work showed that from the nine strains of potentially drought-tolerant bacteria evaluated, three were found to be efficient strains under drought for the studied genotype. One isolated from common bean productive plants under drought conditions, 353 (*Rhizobium gallicum*) and two strains of *Rhizobium etli* isolated from saline soils: A12 and A13. These 3 strains showed high infectiveness (nodule fresh weight) and effectiveness (shoot dry weight) and nitrogen accumulation, even surpassing the plants inoculated with the CIAT899 reference strains in many of the analyzed parameters. In contrast, the strain 343 (*Rhizobium giardinii*) was identified as a sensitive strain, showing the worst results in both water regimes. These results are consistent with the fact that *R. etli* and *R. gallicum* have been previously characterized as microsymbiont that form effective symbiosis with common bean, while *R. giardinii* include both effective and ineffective strains (Ribeiro Torres et al., 2009).

The selected strains (353, A12 and A13) did not present significant differences with the plants chemically fertilized with nitrate neither in the aerial biomass nor in the number of flowers in well-watered conditions and even showed a higher foliar protein concentration. On the other hand, drought affected the N-fertilized plants more intensely, suffering a greater delay in phenological development, and a greater reduction in shoot biomass and number of flowers. Considering that both, the shoot biomass and the number of flowers are parameters closely related to yield (Beebe et al., 2013), the results of the present study showed how through inoculation of plants of Arrocina de Álava with efficient rhizobia strains, yields similar to those obtained with nitrogen fertilization could be obtained in well-watered conditions and surpass them under drought conditions.

The tolerance mechanisms developed by plants of the studied genotype with the inoculation with 353, A12 and A13 strains were a better adjustment of the cell wall elasticity that helps to maintain cellular turgor and volume, prevention of mechanical damages in the plasma membrane, to maintain higher WUE values and to avoid the phenological delay caused by drought developing a greater number of flowers. In further studies we will characterize the selected bacterial strains and delve into the drought tolerance mechanism that underlies this response by evaluating some metabolites related to water stress such as hormone levels, and proline and trehalose content among others. The results obtained provide the basis for the development of “elite” inoculants to increase the yield of an economically interesting genotype common bean under intense drought field conditions in the Northern also covering the need to expand the pool of inoculum resources in Europe (Omari et al., 2022). Therefore, to evaluate the efficiency of these selected rhizobia (353, A12 and A13), a field trial will be carried out under drought conditions. Although the results obtained in the field often differ from those obtained under controlled conditions, very favourable results have been achieved, even with the inoculation of strains with little history of edaphoclimatic adaptation (Sindhu & Dadarwal, 2000; Omari et al., 2022). Thus, taking into account the long tradition of bean cultivation in the Basque Country (Eustat, 2019), that the selected strains were isolated from stressed soils and that common bean is a highly promiscuous species (Shamseldin & Velázquez, 2020), we expect to obtain promising results under field conditions, for Arrocina de Álava genotype, of great economic interest, such as those obtained by other authors with common bean, in other edaphoclimatic zones (Aserse et al., 2020; Rodiño et al., 2021). In future research, we also hope to conduct similar studies with other common bean genotypes, since different genotypes respond differently to the same inoculum (Goyal et al., 2021; Omari et al., 2022).

## 5 Conclusion

From the analysed bacteria, three were found to be highly efficient strains under drought (353, A12 and A13) showing high nodulation capacity, high biological nitrogen fixation and shoot biomass production, even surpassing the plants inoculated with the CIAT899 reference strain, as well as the chemically N-fertilized plants. The tolerance mechanisms developed by inoculated with 353, A12 and A13 strains were, a better adjustment of the cell wall elasticity that prevents mechanical damages in the plasma membrane, higher WUE and avoidance of the phenological delay caused by drought, developing a greater number of flowers. The results confirm that these indigenous strains could have an agronomical application in common bean crops in Northern Spain and be used as biostimulants to reduce negative drought effects related to climate change in a sustainably way, as well as being an alternative to chemical fertilization. Inoculation with these strains could contribute to avoid the environmental N-pollution and costs associated with the production of synthetic fertilizers of great importance nowadays.

## Data Availability Statement

The raw data supporting the conclusions of this article will be made available by the authors, without undue reservation.

## Author contributions

AD-C, AS-S and ML conceived the experiment. AD-C, AS-M, EM, ML conducted the experiment. AD-C, AS-S and ML analyzed data and contributed to the drafting of the manuscript. AD-C wrote the manuscript. AS-S and ML supervised the last version of the manuscript. ML supervised and funded the whole project. All authors contributed to the article and approved the submitted version.

## Supplementary material

Supplementary Figure 1 Leaf biomass (DW, g) of common bean plants nitrate-fertilized (not inoc.) and inoculated with different strains of rhizobium (CIAT899, A5, A9, A10, A11, A12 and A13) under different water availability (WA) conditions: well-watered (blue) and drought (red). When there is no interaction of factors, the multiple comparisons were made comparing all the inoculation treatments with each other, but separating the data according to the water regime (capital letters for control conditions and lower case for drought conditions), and the effect of water availability was studied for each inoculation treatment separately (using asterisks to show the effect): * p< 0.05; ** p<0.01 and *** p<0.001; NS, non-significant).

Supplementary Figure 2 C parameters of common bean plants without inoculum and nitrate-fertilized (not inoc.), and inoculated with different strains of rhizobium (CIAT899, A5, A9, A10, A11, A12 and A13), under different water availability (WA) conditions: well-watered (blue) and drought (red). (* p< 0.05; ** p<0.01 and *** p<0.001; NS, non-significant). **(A)** leaf C concentration, %; **(B)** leaf C total content (mg).

Supplementary Figure 3 Nitrogen content related parameters of common bean plants without inoculum and nitrate-fertilized (not inoc.), and inoculated with different strains of rhizobium (CIAT899, A5, A9, A10, A11, A12 and A13), under different water availability (WA) conditions: well-watered (blue) and drought (red). (* p< 0.05; ** p<0.01 and *** p<0.001; NS, non-significant). **(A)** leaf N concentration, %; **(B)** leaf protein total content (mg).
