# Supplementary material for: Selected indigenous drought tolerant rhizobium strains as promising biostimulants for common bean in Northern Spain
Source: Front Plant Sci. 2023 Mar 29;14:1046397. doi: 10.3389/fpls.2023.1046397 (PMC10090513; doi:10.3389/fpls.2023.1046397)
Supplement: Supplementary file 1 [file Table_1.docx]

Table S.1: Phenological state of common beans at harvest, grown under different water availability (WA) conditions: well-watered (WW) and drought (D).

|  |  | **Number of plants** | | |  |
| --- | --- | --- | --- | --- | --- |
| **WA** |  | **V4** | **R5** | **R6** | **Phenological stage** |
| WW |  | 4 | 8 | 65 | R6 |
| D |  | 18 | 45 | 17 | R5 |
| Total |  | 22 | 53 | 82 | R6 |
