# Supplementary figures and images for: Selected indigenous drought tolerant rhizobium strains as promising biostimulants for common bean in Northern Spain
Source: Front Plant Sci. 2023 Mar 29;14:1046397. doi: 10.3389/fpls.2023.1046397 (PMC10090513; doi:10.3389/fpls.2023.1046397)

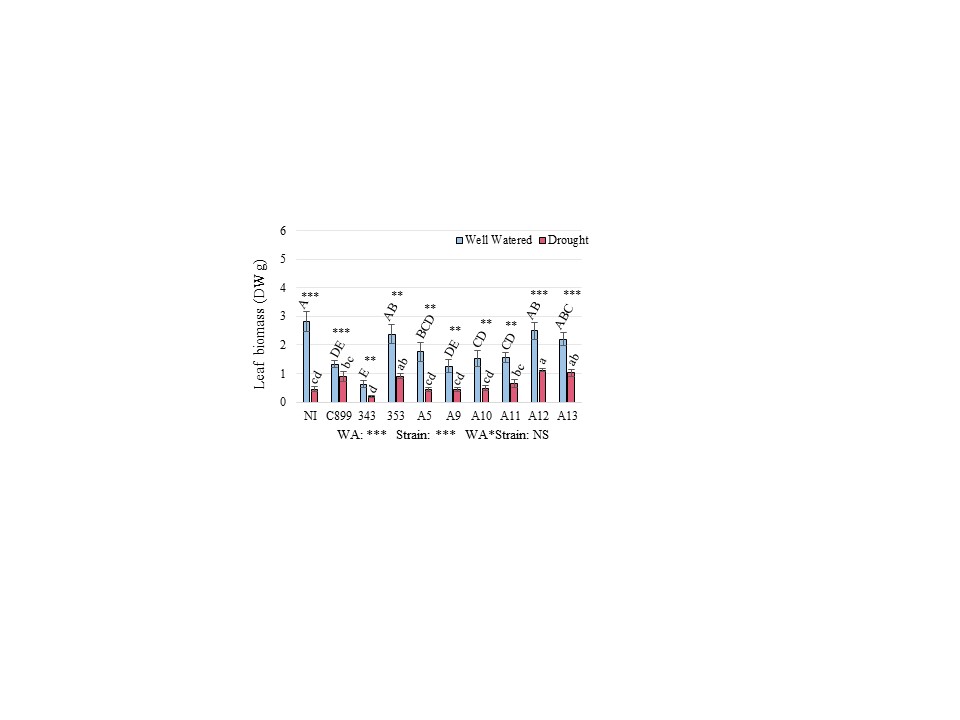

Supplement: Supplementary Figure 1 — Leaf biomass (DW, g) of common bean plants nitrate-fertilized (not inoc.) and inoculated with different strains of rhizobium (CIAT899, A5, A9, A10, A11, A12 and A13) under different water availability (WA) conditions: well-watered (blue) and drought (red). When there is no interaction of factors, the multiple comparisons were made comparing all the inoculation treatments with each other, but separating the data according to the water regime (capital letters for control conditions and lower case for drought conditions), and the effect of water availability was studied for each inoculation treatment separately (using asterisks to show the effect): * p< 0.05; ** p<0.01 and *** p<0.001; NS, non-significant). [file Image_1.jpeg]

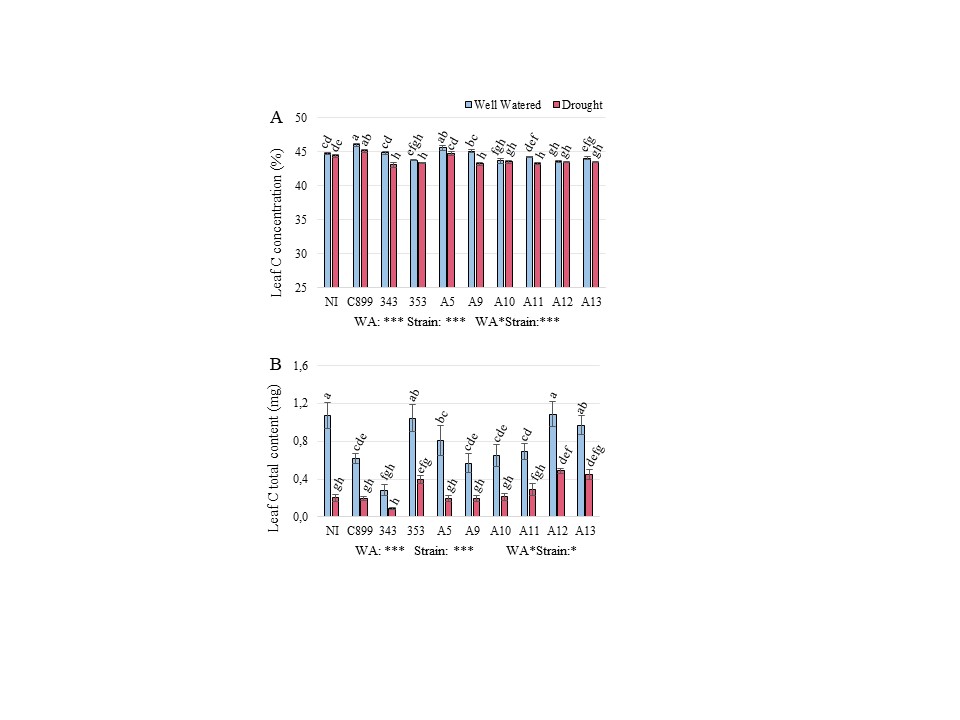

Supplement: Supplementary Figure 2 — C parameters of common bean plants without inoculum and nitrate-fertilized (not inoc.), and inoculated with different strains of rhizobium (CIAT899, A5, A9, A10, A11, A12 and A13), under different water availability (WA) conditions: well-watered (blue) and drought (red). (* p< 0.05; ** p<0.01 and *** p<0.001; NS, non-significant). (A) leaf C concentration, %; (B) leaf C total content (mg). [file Image_2.jpeg]

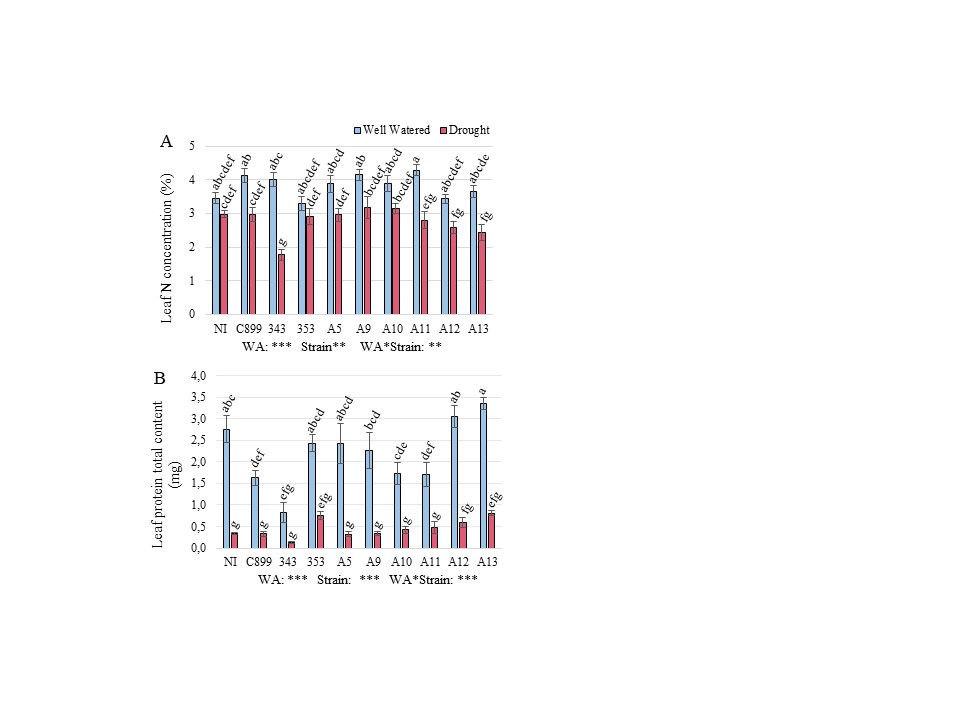

Supplement: Supplementary Figure 3 — Nitrogen content related parameters of common bean plants without inoculum and nitrate-fertilized (not inoc.), and inoculated with different strains of rhizobium (CIAT899, A5, A9, A10, A11, A12 and A13), under different water availability (WA) conditions: well-watered (blue) and drought (red). (* p< 0.05; ** p<0.01 and *** p<0.001; NS, non-significant). (A) leaf N concentration, %; (B) leaf protein total content (mg). [file Image_3.jpeg]
